# Do current methods of measuring the impact of chronic pain on work reflect the experience of working-age adults? An integrated mixed-methods systematic narrative review

**DOI:** 10.1097/j.pain.0000000000003169

**Published:** 2024-02-20

**Authors:** Anne L. Stagg, Ira Madan, Nicola Fear, Martin J. Stevens, Elaine Wainwright, Jan L. Hoving, Gary J. Macfarlane, Rosemary Hollick, LaKrista Morton

**Affiliations:** a Versus Arthritis/Medical Research Council Centre for Musculoskeletal Health and Work (Guy's & St Thomas' NHS Foundation Trust and King's College London), London, United Kingdom; b Versus Arthritis/Medical Research Council Centre for Musculoskeletal Health and Work (King's Centre for Military Health Research, King's College London), London, United Kingdom; c Versus Arthritis/Medical Research Council Centre for Musculoskeletal Health and Work (University of Aberdeen), Aberdeen, United Kingdom; d Department of Public and Occupational Health, Cochrane Work, Amsterdam UMC, University of Amsterdam, Amsterdam, the Netherlands

**Keywords:** Chronic pain, Work, Questionnaire, Development, Mixed-methods review

## Abstract

Chronic pain affects individuals' work participation. The impact of chronic pain on work has historically been measured through sickness absence, though it is now appreciated that the impacts on work are far wider. This mixed-methods review aimed to identify the full range of impacts of pain on work in addition to impacts that are currently measured quantitatively to inform the development of a new questionnaire assessing the wider impacts of chronic pain on work. MEDLINE, Embase, PsychINFO, and CINAHL were searched for studies that included quantitative measures of the impact of chronic pain on work and for qualitative studies where individuals described impacts of their chronic pain on work. Quantitative measures, and text from qualitative studies, were analysed thematically. A thematic framework was developed for establishing the types of impacts measured or described in the literature. Forty-four quantitative and 16 qualitative papers were identified. The literature described impacts within 5 areas: changes at work and to working status; aspects of the workplace and work relationships; pain and related symptoms at work; psychological factors; and factors and impacts outside the work environment related to work. Quantitative measures mainly assessed impacts related to the quantity and quality of work (29 of 42 measures). Seventeen aspects were only discussed within the qualitative literature. This study identifies a discrepancy between the impacts that have been the focus of quantitative measures and the range that individuals working with chronic pain experience and highlights the need for a new measure assessing a wider range of issues.

## 1. Introduction

Approximately 1 in 5 people experience moderate-to-severe chronic pain.^9^ It affects physical and mental health and limits participation in family and social life.^22,29^ Chronic pain also limits participation in paid work,^45^ and work participation is important for individuals: it increases self-worth, creates a sense of purpose and role in society, as well as enabling financial independence.^7^ Unemployment increases pain, worsens mental health, and limits life expectancy.^41^

The impact of chronic pain on work participation was originally measured through sickness absence, although now it is appreciated that there are wider impacts.^21^ It may affect how efficiently and effectively someone does their job (ie, their productivity), and in one early study of chronic health conditions at a major chemical company in the United States, the cost of productivity loss greatly exceeded the cost of absenteeism and medical care combined.^10^ In the medium term, chronic pain can also affect career options and choices and therefore influences the type of work (and financial reward) which someone with chronic pain can undertake.

However, measuring the impact of chronic pain on work has proved challenging. A review of measures of presenteeism (at-work productivity loss due to ill health or other medical conditions^21,27^) found that “self-reported presenteeism instruments have become more differentiated and complex by incorporating many different contextual factors that may impact levels of presenteeism” but that there was a need for future instruments to be underpinned by robust empirical research.^27^ Measures currently available tend to assume someone has regularly contracted work. However, today we work in very different ways—people can have more than one paid employment, while others will be employed on “zero-hour” contracts (3.4% of UK employees) or be both self-employed and in paid employment.^1,16^ These types of modern contracts come at the cost of workers' entitlements to paid leave and sickness absence, and workers are uncertain about income week-to-week. These workers may be vulnerable to ill health not only through the nature of their work, but also through the uncertainty of available work, lack of training, and lack of health surveillance at work.^5^ Not only is the impact of “modern” work unknown, but available instruments make assumptions that people work full-time in one job with regular hours and therefore the current instruments will often not be relevant or ask questions in a way that makes no sense to modern workers.

The aim of the Quantifying the Impact of Chronic pain on paid worK (QUICK) study is to develop an instrument that can be used to assess the impacts of chronic pain on work ability considering the range of ways in which people now engage in paid work. This review comprises the first part of the QUICK study and its aims are to (1) identify how impacts of chronic pain on engagement in work have been measured quantitatively; (2) understand, from the perspectives of individuals with experience of working with chronic pain, how their pain has affected work; and (3) contrast the range of impacts identified quantitatively with those identified qualitatively. By using the qualitative literature to identify impacts of the chronic pain experience, which are not currently measured quantitatively, this review will provide key information to facilitate the development of a new comprehensive measure for the impact of chronic pain on work.

## 2. Methods

Our review was guided by the methodological advice for conducting mixed-methods systematic reviews and the adapted PRISMA (Preferred Reporting Items for Systematic reviews and Meta-Analyses) for reporting systematic reviews of qualitative and quantitative evidence where quantitative results are combined with the results from the qualitative literature to identify overlaps and gaps in the literature.^24,47^

### 2.1. Search strategy

A comprehensive list of search terms reflecting “chronic pain” and “work” was developed including using published search terms from systematic reviews reporting terms for work participation.^12,17,25,28,33,37,46^ A specific list of conditions that are commonly associated with chronic pain was also developed. These conditions included both inflammatory and noninflammatory rheumatic and musculoskeletal conditions and other pain-related conditions (eg, painful skin conditions, chronic headache, and pelvic pain). Studies identified based on these conditions were only included if chronic pain status was measured and confirmed separately (and not inferred by the presence of a condition).

The search was conducted using Embase, Medline, Psych INFO, and CINAHL. Limits were applied on the electronic database searches to exclude reviews and meta-analyses and articles not published in English. The search time frame was limited from 2010 to the search date (21 July 2021) to enable the identification of current measures of the impact of chronic pain on work. The full search strategy used in Medline is listed in Supplementary File Table 1, http://links.lww.com/PAIN/C5. This was adapted for use in each database. Additional studies were identified by consulting with experts in the field of employment, health, and chronic pain, and from examining reference lists of relevant systematic reviews and articles included in the final analysis.

### 2.2. Screening

Search results were uploaded to Covidence systematic review software (www.covid ence.org, Veritas Health Innovation Ltd, Melbourne, Australia). Duplicates were removed before screening of titles and abstracts. Studies selected for full-text screening were then exported to Endnote and assessed for eligibility. At both stages of screening, at least 10% of references were dual screened and any discrepancies discussed and resolved by consensus. Quantitative studies using measures that were not freely available online were excluded.

### 2.3. Inclusion and exclusion criteria

Quantitative and qualitative articles were included. Full inclusion and exclusion criteria are reported in Table 1. Taken together, these criteria facilitated the identification of studies where:

(1) A quantitative measurement tool or item was used to assess a work/employment–related impact within a sample of individuals with chronic pain or
(2) Individuals described the impact of chronic pain on participating in employment or reflected more widely on the ability to engage in work.

**Table 1:** Inclusion and exclusion criteria.

An article was included if all the following criteria were met:
Participants: adults between 16 and 65 y or where average age of participants was younger than 65 y (male UK state pension age in 2010 was 65 y)
Study design: original research
Pain: studies where chronic pain (3 mo or more) was recorded
Work/employment: clear focus on the impact of chronic pain on work/employment where individuals with chronic pain were either employed and working or on temporary or longer-term sick leave due to chronic pain but aiming to return to work
For quantitative studies, the impact of chronic pain on any aspect of work participation was measured either using a questionnaire in its entirety, specific items taken from a questionnaire, or where a study used novel question(s) that were published in full
For qualitative studies, individuals described the impact of chronic pain on an aspect of their work or employment
An article was excluded if any of the following criteria were met:
Pain: pain due to injuries or postsurgery procedure
Work/employment: the only information collected about employment was work status

### 2.4. Data extraction

#### 2.4.1. Quantitative studies

The following data were extracted from each included article: reference details; information on the presence of pain and condition; employment context; details of the instrument/item(s) used to assess the impact of pain on work; and details of impacts that were measured.

#### 2.4.2. Qualitative studies

Eligible qualitative studies were exported to NVivo 1.5.1 software for separate thematic analysis with information extracted on the presence of pain and condition, employment context, and impacts described.

### 2.5. Analysis

Included articles were described in terms of their populations' characteristics and employment context. To integrate quantitative results with the qualitative themes, the quantitative findings were *qualitised.* Qualitisation has been defined as *extracting data from quantitative studies and translating or converting it into textual descriptions*.^47^ In the context of this study, quantitative work-related instruments/items were categorised and described qualitatively in terms of the types of impact(s) they assessed. This approach facilitated the collation of work-related impacts that have been used in recent quantitative research and how they have been measured.

Results sections within the included qualitative studies were coded line by line for content regarding any aspect of work affected by chronic pain. Coded content included both that which was written as summary results and direct quotations within the studies.

#### 2.5.1. Development of the thematic framework

**Figure 1.**
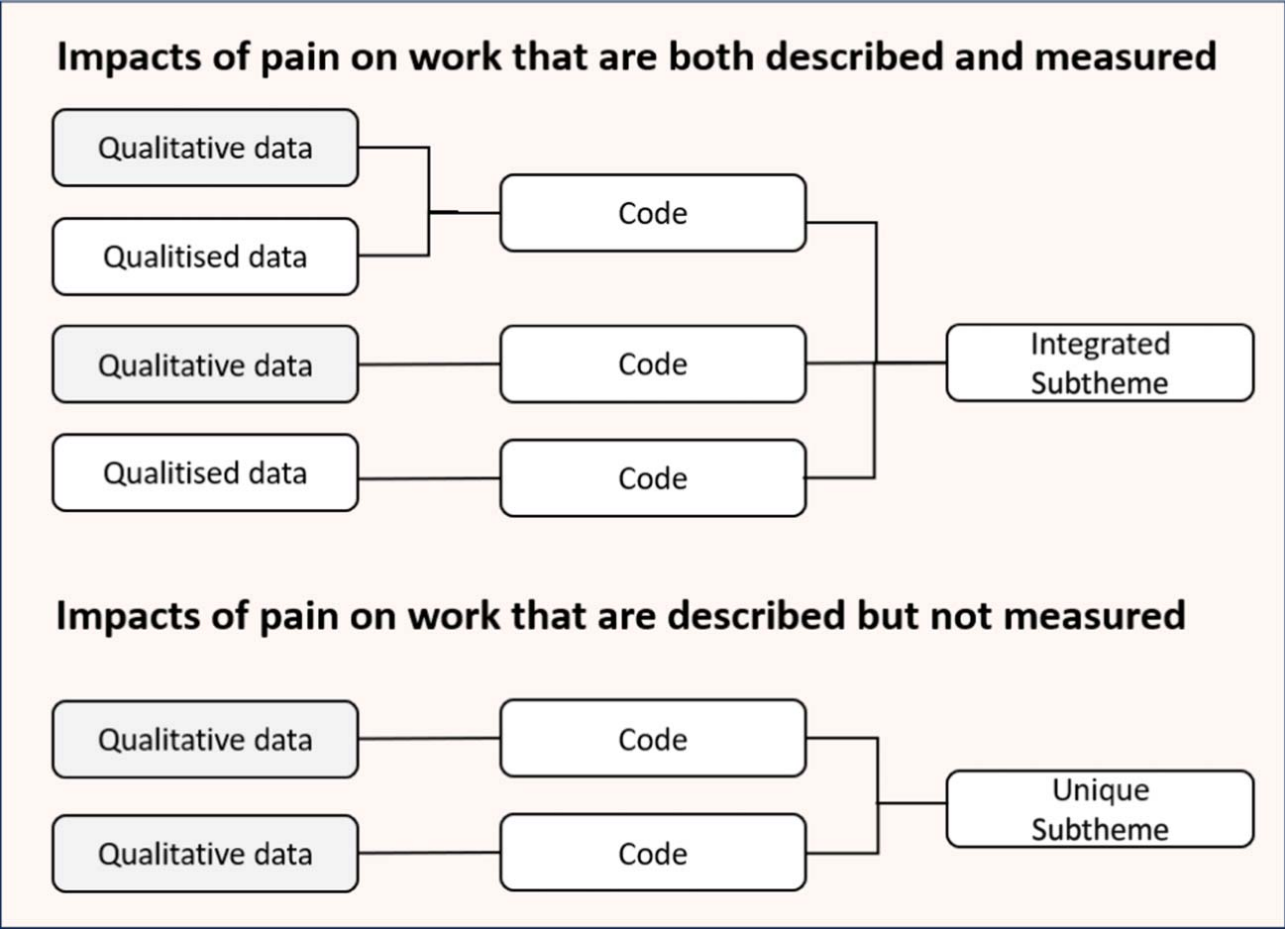
Thematic framework. Summary of the convergent integrated approach used to develop the thematic map that identified unique subthemes in qualitative data with no corresponding quantitative measure. Qualitised data refer to impacts of pain on work that are measured in quantitative studies.

Impacts of pain measured in quantitative studies and described by individuals with experience of working with chronic pain in the qualitative studies were combined within a thematic framework. To facilitate the comparison between quantitative and qualitative analyses, the themes used to categorise impacts were developed iteratively and in parallel between the quantitative and qualitative studies using a convergent integrated mixed-methods approach.^47^ The integrated thematic framework provided a description of impacts that were either (1) described qualitatively and measured quantitatively, (2) only described qualitatively, or (3) only measured quantitatively. This method of synthesis enabled gaps in current measures of impact (ie, only identified in the qualitative literature), to be identified and characterised at subtheme level, as summarised in Figure 1. Risk of bias of included articles was not assessed given the scope of the review was to identify measures and items used to measure the impact of chronic pain on work, rather than assess the quality of any research that used these measures to investigate a particular research question.

## 3. Results

### 3.1. Study selection

**Figure 2.**
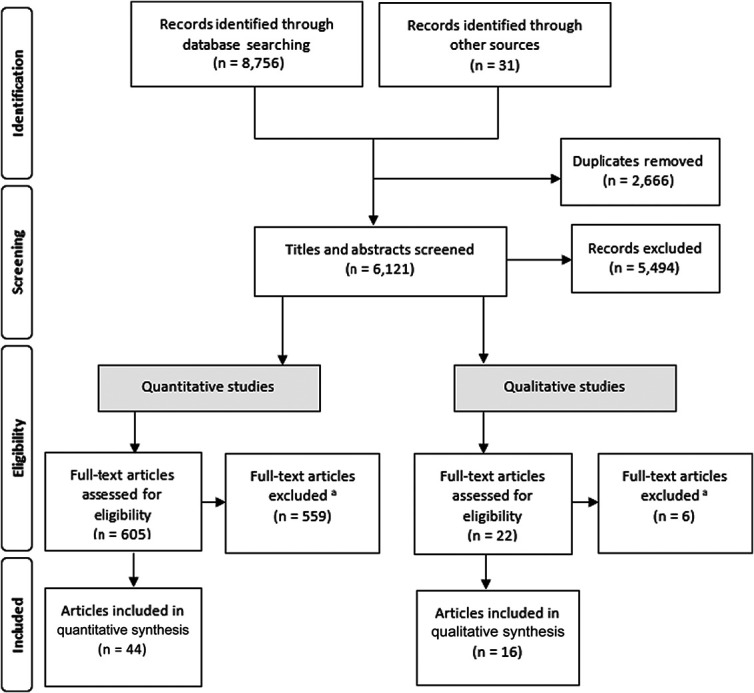
PRISMA diagram of included articles. ^a^Quantitative articles reasons for exclusion: 555 did not meet 1 or more of the inclusion criteria of measured chronic pain, a contextual link between chronic pain and engaging in work, and the impact of pain on work was measured. Four articles were excluded because questionnaires were not freely available. Qualitative articles reasons for exclusion: 3 = not chronic pain, 1 = a qualitative review, 2= focusing on the effectiveness of interventions and not chronic pain. PRISMA, preferred reporting items for systematic reviews and meta-analyses.

Database searches and other sources identified 8787 studies. After removal of duplicates (n = 2666), 6121 references were screened for inclusion at title and abstract stage, and 605 quantitative and 22 qualitative articles were subsequently selected for full-text screening. There were 44 quantitative and 16 qualitative articles included in the final data extraction. The full PRISMA flow diagram is provided in Figure 2.

### 3.2. Study characteristics

The quantitative articles represented studies conducted in Europe (with 1 European study including the UK^40^) (n = 22), North America (n = 15), Australasia (n = 3), the Middle East (n = 1), and multiple geographic areas (n = 3). Qualitative studies were conducted in Europe (n = 9), including 2 in the UK,^31,51^ North America (n = 5), Australasia (n = 1), and South America (n = 1).

Within eligible articles, participants represented a range of chronic pain conditions and employment contexts (Table 2). Most quantitative articles reported on a measure related to chronic pain and work within the context of individuals with back pain (n = 14) or other musculoskeletal pain (n = 14) and reflected samples of individuals who were employed (n = 23) with 3 of these studies focusing on individuals who were on sick leave or at risk of sick leave due to chronic pain. Qualitative articles generally reported on individuals with musculoskeletal pain (n = 10) and reported on samples of individuals who were describing impacts of their pain on work retrospectively because they were not working during the study (n = 7). Full details of each included quantitative articles are summarized in Supplementary File Table 2, http://links.lww.com/PAIN/C5 and of each included qualitative article in Supplementary File Table 3, http://links.lww.com/PAIN/C5.

**Table 2:** Summary pain and employment characteristics for included eligible articles.

	Quantitative studies *n* (%)	Qualitative studies *n (%*)
Chronic pain context		
Back pain	14 (32)	3 (19)
Other musculoskeletal pain	14 (32)	7 (44)
Chronic pain (nonspecified/mixed)	7 (16)	3 (19)
Fibromyalgia	3 (7)	2 (13)
Pelvic pain	3 (7)	0 (0)
Neuropathic pain	2 (5)	0 (0)
Migraine	1 (2)	0 (0)
Chronic widespread pain	0 (0)	1 (6)
Employment context		
All employed (including full-time, part-time, and self-employed)	20 (45)	2 (13)
Employed but on sick leave or at risk of sick leave	3 (7)	4 (25)
Mixed (employed/on sick leave/unemployed/unable to work (disabled)/retired)	12 (27)	6 (38)
Unemployed/unable to work (disabled)	0 (0)	3 (19)
No baseline employment status reported	9 (20)	1 (6)

### 3.3. Impacts of chronic pain on work within the integrated thematic framework

The literature described aspects related to impacts of chronic pain on work within 5 key areas (Table 3). Changes to work and working status included job changes and adjustments, reduced productivity both at work (presenteeism) and due to sick leave (absenteeism), and factors relating to returning to work. Aspects of the workplace and work relationships included facilitators at work such as having supportive employer and colleagues and job control, so that adjustments can be made. By contrast, this theme also included lack of support from employers and lack of control being barriers to engaging in work. Being unable to be open with an employer or colleagues (issues with disclosure) was an additional barrier. This code also included perceived benefits of work for those with chronic pain such as work providing a structure and routine each day and a “normal” social setting. The third theme was the direct impact of pain and symptoms on work, with pain-related fatigue and physical work potentially aggravating the pain. The irregular nature of pain affecting consistent engagement in work was also included in this subtheme. However, there was also a positive aspect, with engaging work providing a distraction from the pain. A major theme was the psychological impacts of pain on work, which had 4 subthemes. These included negative psychological impacts on cognition and emotional exhaustion, work-related stress, negative beliefs about working, expectations about unemployment, and fear of movement. However, also included were psychological factors that can have a positive influence on engaging in work such as self-efficacy, having a positive attitude towards work, and benefits of work including it providing a sense of purpose and value. The fifth theme relates to impacts on engagement in work that are relevant, or occur, outside the workplace such as reduced household income and positive impacts linked to work-related support, for example, from health care professionals, family, and friends.

**Table 3:** Comparison of themes, subthemes, and codes in the quantitative and qualitative literature.

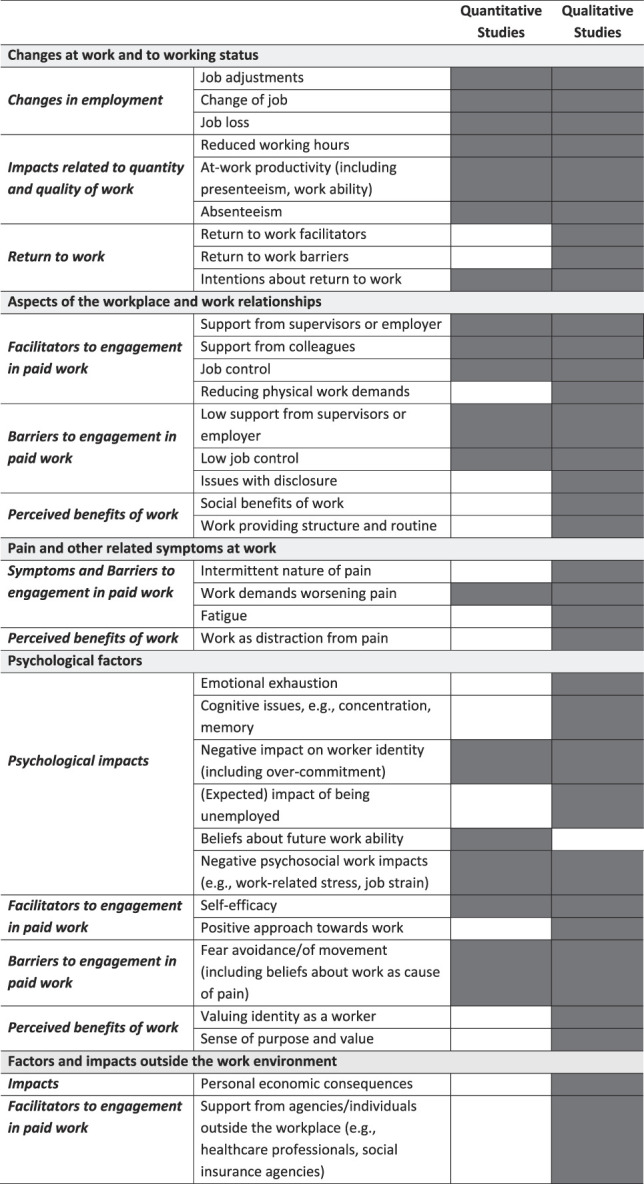

### 3.4. Quantitative items and instruments

Twenty-one questionnaire instruments or subsets of items drawn from these instruments and 21 stand-alone quantitative item sets (or individual items) assessed 1 or more aspects within 4 of the themes (Table 3). The Work Ability Index (WAI), Work Ability Limitations Scale (WALS), and Work Instability Scale (WIS) were the only 3 questionnaires that were solely focused on assessing an impact of chronic pain on work, and these all measured at-work productivity.^2,8,14,40^ The total number of instruments and items that were mapped to each of the key themes, and more detailed subthemes, are listed in Table 4. Instruments and items frequently focused on assessing “impacts related to quantity and quality of work,” primarily measuring presenteeism, absenteeism, and work ability (n = 29). By contrast, all other impacts were assessed by 5 or fewer item sets/instruments. Four studies assessed 5 psychological impacts including work-related stress, psychological demands of a job, and work ability expectations,^2,15,19,50^ and 4 studies assessed changes in employment, which included changing working hours, job adjustment, sick leave, job loss, and retirement.^2,4,6,49^ Considering the work contexts of quantitative studies, most reported on participants who were employed either full-time or part-time (n = 20) with only one of these studies recording 14.5% of their study population being self-employed but no further information regarding the type of self-employment. Twelve studies reported on participants in a mixture of work contexts including those who were unemployed, unable to work, and retired. There was no reference to types of employment contracts (eg, zero hours contracts) in any of the quantitative studies. In addition, the items in 2 of the measures used in their entirety, (the WALS and the WIS), assume that the participant is in paid employment with little control over their job because they contain items questioning the ability to manage to work the hours that the job demands and using holiday leave to avoid having to go off sick. Details of each included instrument and item set are included within Supplementary File Table 4, http://links.lww.com/PAIN/C5.

**Table 4:** Number of quantitative instruments and items that map to each aspect within the thematic framework.

Themes	Sub-themes	Count of quantitative item sets/instruments
Changes at work and to working status	Changes in employment Impacts related to quantity and quality of work Return to work	4 29 2
Aspects of the workplace and work relationships	Facilitators to engagement in paid work Barriers to engagement in paid work Perceived benefits of work	2 2 0
Pain and other related symptoms at work	Symptoms and barriers to engagement in paid work Perceived benefits of work	1 0
Psychological factors	Psychological impacts Facilitators to engagement in paid work Barriers to engagement in paid work Perceived benefits of work	5 1 1 0
Factors and impacts outside the work environment	Impacts Facilitators to engagement in paid work	0 0

### 3.5. Impacts in the qualitative literature

A range of nuanced impacts and aspects related to impacts of chronic pain on work were described within the qualitative literature. Within the overall thematic framework that was developed from both the quantitative and qualitative literature, all themes and subthemes were discussed within the qualitative literature (detailed in Supplementary file Table 3, http://links.lww.com/PAIN/C5 with an illustrative quotation from each of the key themes listed in Table 5). Participants with experience of working with chronic pain described the importance of their role and the context of their employment setting, including relationships with colleagues and employers, in shaping the impacts they experienced. Complex relationships were often described, which illustrated the multifaceted nature of impacts related to chronic pain at work, where one impact can lead to a series of further negative impacts. For example, in the second quote within Table 5, the individual describes a reluctance to disclose their pain to their manager and this in turn affects the ability to access any job accommodations; as a consequence, their life outside of work and their medication use to manage their pain are affected. However, benefits of work within several of the key themes were also identified. Within the fourth quote in Table 5, the individual describes how work is perceived to distract them from their pain. Social and psychological benefits of work, for example, valuing one's identity as a worker, were also described within the context of their chronic pain. Of importance, these benefits (as well as the impacts described) were described concurrently with descriptions of their pain or within the context of being an individual with pain, ie, they were linked to their pain experience rather than described separately from it.

**Table 5:** Themes from the qualitative literature with example quotes.

Theme	Example quotations (subtheme: code)
Changes at work and to working status	*I was on morphine, you could see I was glassy eyed, the company tries to be accommodating by saying, “Oh we can give you a position near your workplace, near your home, near your treatment centers or a position that won't require physically demanding tasks.” I was a bank teller, I would have to stand all day, and after 3 or 4 months they're like, “We can't find anything so we're just gonna let you go.”*^48^ (Changes in employment: job loss)
Workplace and work relationships	*I don´t want to say to my manager that I can´t manage this, that I need some help, so I try and I clench my teeth and I ache all over…but I do the job and when I come home I collapse and take strong medication for the night to manage the next day*.^30^ (Barriers to engagement in paid work: issues with disclosure)
Psychological factors	*How I once defined myself, called myself, I don't have that definition of self any more. So for me that's a scary thing because…I always thought of myself a certain way and so now I don't know who I am or what I am, what my role is…sort of losing that sense of self. I'm not the same person that I was 5 years ago and I never will be. And I don't know who I'll be or what I'll become or what I can do. So for me that's the biggest thing. It's just loss of self definition.*^44^ (Psychological impacts: negative impact on worker identity)
Pain and other related symptoms at work	*When I sit at home in pain, I get so isolated in my head by my pain. Getting out of the house, being at work among healthy people is enough to be able to forget that I have a problem. There (at work) I suddenly become a healthy person again. I can throw this crap behind me and be healthy for a while.*^36^ (Perceived benefits of work: work as distraction from pain)
Factors and impacts outside the work environment	*It is not only a question of what salary I get today, it's also about my pension and a lot of things*.^36^ (Factors and impacts outside the work environment: personal economic consequences)

### 3.6. Comparison of quantitative and qualitative literature

The codes listed in Table 3 illustrate the range of specific issues related to the impacts of chronic pain on work experienced by individuals. The theme of *factors and impacts outside the work environment* was only reported in the qualitative literature, with no measures identified quantitatively relating to the personal economic consequences of pain or the positive impact on engagement in work linked to support from outside the workplace. In addition to this theme, 5 subthemes were also not assessed by any quantitative measures. These subthemes primarily focused on the perceived benefits of work within the context of the workplace and workplace relationships, positive impacts of work on pain and symptoms, and beliefs about the perceived benefits of work. There were 10 further codes within 6 additional subthemes that were not assessed quantitatively but were discussed within the qualitative literature. These codes included factors related directly to pain-related symptoms and negative psychological impacts of pain such as the intermittent nature of pain, fatigue, emotional exhaustion, and resulting difficulties with concentration and memory at work. Whilst return to work was a subtheme in the thematic framework, the obstacles and facilitators of returning to work were not assessed in the quantitative literature whilst being reported in the qualitative literature. Only intentions about returning to work in the future was assessed quantitatively.

In contrast to the multiple gaps identified in quantitative measures, there was only 1 code not discussed within the qualitative literature that had been assessed quantitatively (*beliefs about future work ability*), although a similar code, *intentions about returning to work,* was reported in the qualitative literature and coded in the *return to work* subtheme.

There was, however, concordance between quantitative and qualitative studies in the codes and subthemes comprising the theme *changes at work and to working status*. The existence of the fear avoidance beliefs questionnaire (and used in 6 quantitative studies)^18,19,26,32,39,50^ resonates with the descriptions of fear of movement affecting engagement in work that was reported in the qualitative literature.^3,30,34,35^ Other impacts that were integrated qualitative/qualitised codes included *support from supervisors or employers* and *job control* and were reported in terms of both being positive impacts when present and negative impacts when absent. Finally, as a facilitator to engagement in work in the context of chronic pain, self-efficacy was reported extensively in qualitative literature^35,36,42,43^ but was assessed quantitatively by a single stand-alone item.^20^

## 4. Discussion

This mixed-methods systematic review found that current quantitative measurements of the impact of chronic pain on work are limited in comparison with the overall experience described by individuals in qualitative studies.

Most quantitative studies in our search focussed on measuring impacts related to productivity loss (eg, presenteeism and absenteeism), whereas the qualitative studies described a wider range of influences and interrelationships which individuals with chronic pain consider to be important factors in whether, and how, they participate in work. These include the impacts of pain, fatigue, emotional exhaustion, concentration difficulties, the availability of job adjustments, and the degree of openness in communication with managers. The findings also indicate positive aspects of engaging in work that are relevant to individuals with chronic pain but are not assessed by current instruments.

Our findings reflect those of a large systematic review of work participation outcomes in randomised controlled trials (RCTs) across disciplines, which was undertaken as part of a project to develop a core outcome set for work participation.^38^ Similar to our review, outcome measures relating to changes at work and working status comprised the largest number of work outcome measures (n = 398, 91.5%). In comparison, this review identified 35 measures within this theme (74.5%). The Ravinskaya review developed a different classification framework comprising 4 themes, with 3 of these being related to our theme of “changes at work and to working status” (“employment status,” “absence from work and return to work,” and “at-work productivity loss”), and 1 additional theme of “employability” relating to gaining and maintaining employment. Employability also included 3 measures of self-efficacy, which we categorised differently as a psychological factor within the subtheme of “facilitators to engagement in paid work.” Our review identified a wider range of constructs because it had additional specificity in relation to chronic pain and was not limited to identifying measures used within an RCT context. In doing so, in addition to the unique qualitative impacts included within our framework, we were able to identify existing quantitative measures and items for psychological and symptom-related impacts to engagement in work, as well as aspects of the workplace, workplace relationships, and job control specifically related to the impact of chronic pain on work.

We are not aware of any other systematic review that has investigated the wider impacts of pain on engaging in work or any review which has included both quantitative and qualitative studies. A systematic review investigating the impact and burden of chronic pain in the workplace took an employer-focused outcome approach, rather than employee-focused outcome approach, reporting only absenteeism (sickness absence), presenteeism, and employment status.^37^ It is clear from the findings of our review that including qualitative evidence has added to knowledge of additional contextual impacts that can inform a new measure with the aim of providing more a comprehensive understanding of the impacts of pain on work. Similarly, Hollick et al.^23^ used mixed methods to compare presenteeism, absenteeism, and overall work impairment in patients with axial spondylarthritis living in urban and rural settings. Whilst increased presenteeism in rural areas could be explained by disease activity (as measured by quantitative instruments), patient interviews provided far greater insights into factors that affect engaging in work.^23^ Several of the findings of the Hollick study are reflected in our review; a range of factors such as the degree of job control and work providing a positive sense of self-identity as well as negative impacts of pain leading to fatigue and related cognitive issues are implicated in work participation for individuals with painful conditions.

A strength of our review was the inclusion of both quantitative and qualitative studies and the comprehensive nature of our search. However, some methodological limitations should be acknowledged. During the literature search phase of the review, some studies that might have provided additional information were excluded because of the strict exclusion criteria that were applied. For example, studies were excluded if presence of chronic pain was not specifically assessed. This was particularly evident in studies where participants had a diagnosed condition associated with pain (eg, rheumatoid arthritis or fibromyalgia) but where pain was not assessed separately from the primary condition. However, the pain contexts included in the analysis were diverse, covering a wide range of conditions associated with chronic pain, such as chronic migraine, cancer pain, endometriosis, fibromyalgia, neuropathic pain, as well as arthritis and musculoskeletal pain.

A further limitation of our review is that measures that were not freely available, or in English, were not included, which may have led to exclusion of some relevant items. However, whilst we were unable to access the specific items within several nonaccessible measures, the narrative descriptions of these measures' constructs within the included studies indicated that the impacts assessed would not have altered the findings of the current study. For example, one study using the Dutch questionnaire on the Perception and Evaluation of Work used this measure to report job control and social support at work from supervisors and coworkers, and these constructs were identified within the theme of “aspects of the workplace and work relationships” in this study.^13^ Similarly, 2 studies used the Job Content questionnaire (JCQ) to assess social support at work from supervisors and coworkers, although without access to the details of the items within the JCQ, they were not included in the analysis.^2,11^ However, one study used the JCQ to report “psychological demands,” and without access to the details of the items within the JCQ, we were unable to assess which psychological aspects were measured and whether they had already been identified by our analysis from other studies.^11^

It must also be acknowledged that the impacts identified in this study are derived from research conducted within the developed world and may limit the relevance of a new measure to work settings within this context. It is important that further research is conducted in developing countries so that the range of work impacts in these different contexts can be established.

Given the common nature of chronic pain in working populations, it is important that policies and interventions that support working with chronic pain are applicable to as wide a range of people as possible, and therefore the way in which impacts are established and addressed are kept as generalisable as possible. Such generalisability will also reduce the need for numerous work impact questionnaires and facilitate comparative research studies across multiple pain-related conditions. Furthermore, from a methodological perspective, a quantitative instrument that includes a diverse range of impacts has the potential to enable more efficient and insightful delivery of services and, particularly, increase the efficiency of mixed-method studies. For example, by including a robust instrument that focuses on impacts that are important to people working with chronic pain, complementary qualitative inquiries could focus on understanding nuanced or situation-specific contextual factors, which may contribute to, or be barriers or facilitators in the management of, these impacts. This approach could in turn support the selection of appropriate and timely interventions for a given population. Furthermore, applications of a new tool, beyond assessing the impact of chronic pain on work in a given setting at a specific point in time, could include the assessment of changes at an individual level (eg, based on changes to approaches to management), at group level (eg, through workplace interventions), and through population approaches (eg, through policy changes).

In summary, our findings support the development of a new measure for the impact of chronic pain on work that can be used in diverse occupational and clinical contexts. This study provides key information on impacts not measured in current tools, which should be considered for inclusion. Multidimensional impacts, illustrating the relevance of the workplace context, relationships at work, personal psychological factors, and wider impacts outside the work environment are pertinent to the development of a new quantitative measure, with additional qualitative work to ascertain the importance of the range of impacts identified.

## Conflict of interest statement

The authors have no conflict of interest to declare.

## Appendix A. Supplemental digital content

Supplemental digital content associated with this article can be found online at http://links.lww.com/PAIN/C5.

## Supplementary Material

SUPPLEMENTARY MATERIAL
