# Supplementary material for: Do current methods of measuring the impact of chronic pain on work reflect the experience of working-age adults? An integrated mixed-methods systematic narrative review
Source: Pain. 2024 Feb 20;165(7):1472–81. doi: 10.1097/j.pain.0000000000003169 (PMC11190898; doi:10.1097/j.pain.0000000000003169)
Supplement: SUPPLEMENTARY MATERIAL [file jop-165-1472-s001.pdf]

Supplementary File

Supplementary File Table 1. Full search strategy used in Medline, adapted for use in other databases

#	MEDLINE	21 Jul 2021
1	work.mp. or exp Work/ or exp Employment/ or employment.mp. or job.mp. or occupation.mp. or exp Occupations/ or vocation.mp.	1,332,138
2	employment status.mp. or exp Employment/ or work status.mp. or job status.mp.	98,455
3	((work and social adjustment scale) or WSAS or WHODAS or WHO disability assessment schedule or world health organisation disability assessment schedule or HPQ or (world health organization health and work performance questionnaire) or WAI or work ability index or WPAI$ or WPAI or (Work productivity and activity impairment questionnaire) or QQ or quality quantity questionnaire or WALS or workplace activity limitations scale or WLQ$ or work limitations questionnaire$).mp.	4,068
4	productivity.mp. or exp Efficiency/ or efficiency.mp. or work engagement.mp. or exp Work Engagement/	566,703
5	(work.mp. or exp Work/ or job.mp. or employ$.mp. or occupation$.mp.) and (participation or function$ or attend$ or ability).mp.	459,304
6	presenteeism.mp. or exp Presenteeism/ or (reduc$ adj3 work$ adj3 perform$).mp. or ((worktime or work time or workday$ or work$ day$ or productivity) and los$).mp.	12,618
7	(((work or job) and (disab$ or impair$ or incapacity or limitation or instability)) or occupation$ stress).mp. or exp Occupational Stress/	89,608
8	(disab$ management or work capacity evaluation).mp. or exp Work Capacity Evaluation/ or ((work or job) adj2 (modif$ or adjust$)).mp. or ((employment or job) adj2 support$).mp. [mp=title, abstract, original title, name of substance word, subject heading word, floating sub-heading word, keyword heading word, organism supplementary concept word, protocol supplementary concept word, rare disease supplementary concept word, unique identifier, synonyms]	11,481
9	absenteeism.mp. or exp Absenteeism/ or ((sick$ or illness or disability) adj2 (leave or day$ or absen$)).mp. or medical leave.mp. or (sicklist$ or sick list$).mp.	24,342
10	((long term and (absen$ or sick$)) or ((duration or work) adj3 absen$) or ((disab$ or sick$) and (benefit$ or pension))).mp.	68,432
11	(((work$ or job) and retention) or ((stay or remain) adj at work)).mp.	21,606
12	(((return$ or resum$ or re-integrat$ or reintegrat$ or re-entry or reentry or reenter$ or re-enter or recommence) adj2 (work or employment or labo?rforce or labo?r-force or labo?r market$)) or return to work).mp. or exp Return to Work/ or reemploy$.mp. or re-employment.mp. or work resumption.mp. or rehire$.mp. [mp=title, abstract, original title, name of substance word, subject heading word, floating sub-heading word, keyword heading word, organism supplementary concept word, protocol supplementary concept word, rare disease supplementary concept word, unique identifier, synonyms]	14,593
13	(work$ intervention or work readiness or ((vocation$ or occupation$ or work$ or job or employ$) and (rehab$ or guidance))).mp. or exp Rehabilitation, Vocational/ or (occupational and (health or intervention or therapy)).mp. [mp=title, abstract, original title, name of substance word, subject heading word, floating sub-heading word, keyword heading word, organism supplementary concept word, protocol supplementary concept word, rare disease supplementary concept word, unique identifier, synonyms]	224,249
14	((job or work or employ$) and loss).mp. or exp Retirement/ or retirement.mp. or early retire$.mp. or (job adj1 change).mp. or unemploy$.mp. or exp Unemployment/	105,905
15	(quality of life or QoL or HRQoL or health related quality of life or (disease adj2 burden)).mp. or exp "Quality of Life"/	405,586
16	exp Health Care Costs/ or cost of illness.mp. or exp "Cost of Illness"/ or health economics.mp. or "Costs and Cost Analysis"/ or (cost adj1 estimate).mp.	143,530
17	or/1-16	2,651,851

18	(chronic adj2 pain$).mp. or exp Chronic Pain/ or persistent pain.mp.	70,571
19	pain.tw.	667,968
20	$arthriti$.mp. or exp Arthritis/ or rheumat$.mp. or exp Rheumatic Diseases/ or spondyliti$.mp. or exp Spondylitis/ or slipped disc.mp. or exp Intervertebral Disc Displacement/ or sciatica.mp. or exp Sciatica/ or exp Carpal Tunnel Syndrome/ or carpal tunnel.mp. or repetitive strain injury.mp. or exp Cumulative Trauma Disorders/ or psoriasis.mp. or exp Psoriasis/ or shingles.mp. or exp Herpes Zoster/ or sickle cell.mp. or exp Anemia, Sickle Cell/ or gout.mp. or exp Gout/ or lupus.mp. or exp Fibromyalgia/ or fibromyalgia.mp. or (chronic adj5 headache).mp. or exp Headache Disorders/ or exp Migraine Disorders/ or migraine.mp. or exp Neuralgia/ or neuralgia.mp. or pelvic inflammatory disease.mp. or exp Pelvic Inflammatory Disease/ or endometriosis.mp. or exp Endometriosis/ or inflammatory bowel disease.mp. or exp Inflammatory Bowel Diseases/ or Crohn disease.mp. or exp Crohn Disease/ or colitis.mp. or exp Colitis/ or exp Somatoform Disorders/ or somatic symptom disorder.mp.	871,533
21	19 and 20	109,082
22	non cardiac chest pain.mp.	502
23	18 or 21 or 22	164,690
24	17 and 23	29,561
25	limit 24 to (english language and full text and humans and yr="2000 -Current" and ("adolescent (13 to 18 years)" or "young adult and adult (19-24 and 19-44)" or "middle age (45 to 64 years)" or "all aged (65 and over)") and journal article)	2,190
26	review.pt.	2,832,204
27	(medline or medlars or embase or pubmed or cochrane).tw,sh.	261,229
28	(scisearch or psychinfo or psycinfo).tw,sh.	43,405
29	(psychlit or psyclit).tw,sh.	916
30	cinahl.tw,sh.	33,064
31	((hand adj2 search$) or (manual$ adj2 search$)).tw,sh.	14,383
32	(electronic database$ or bibliographic database$ or computeri?ed database$ or online database$).tw,sh.	44,501
33	(pooling or pooled or mantel haenszel).tw,sh.	120,189
34	(peto or dersimonian or der simonian or fixed effect).tw,sh.	8,583
35	(retraction of publication or retracted publication).pt.	19,034
36	or/27-35	405,890
37	26 and 36	173,975
38	meta-analysis.pt.	137,806
39	meta-analysis.sh.	137,806
40	(meta-analys$ or meta analys$ or metaanalys$).tw,sh.	236,850
41	(systematic$ adj5 review$).tw,sh.	242,597
42	(systematic$ adj5 overview$).tw,sh.	2,683
43	(quantitativ$ adj5 review$).tw,sh.	8,904
44	(quantitativ$ adj5 overview$).tw,sh.	348
45	(quantitativ$ adj5 synthesis$).tw,sh.	3,488
46	(methodologic$ adj5 review$).tw,sh.	7,137
47	(methodologic$ adj5 overview$).tw,sh.	478
48	(integrative research review$ or research integration).tw.	154
49	or/38-48	377,327
50	37 or 49	443,993
51	25 not 50	**2,136**

Supplementary File Table 2. Summary of quantitative studies measuring the impact of chronic pain on engagement in work

First author, publication year, country) [study reference no.]	Chronic pain context	Employment context	Name of instrument and number of items used, or stand-alone items	Question recall time	Work impact(s) measured
Agaliotis (2013), Australia [3]	Knee pain	All participants in paid employment, mixed job roles.	Absenteeism - 1 item.<br>Presenteeism - 1 item.<br>Both items derived from the WPAI: OA.	Absenteeism: Days off in previous 2 months.<br>Presenteeism: daily diary entries for presenteeism summarised over previous 12 months.	Absenteeism; At-work productivity
Agaliotis (2017), Australia [4]	Knee pain	All participants in paid employment, mixed job roles,	Absenteeism -1 item.<br>Presenteeism - 1 item.<br>Both items derived from the WPAI: OA.<br>Work ability: WAI: 1 item for each of physical and mental demands of the job, reported separately.<br>Work impairment: WALS.<br>Beliefs about future work ability: 1 stand-alone item.<br>Job control: 1 stand-alone item.<br>Work attitude/work through sickness: 1 stand-alone item.	Absenteeism: Days off in previous 2 months.<br>Presenteeism: daily diary entries for presenteeism summarised over previous 12 months.<br>Work impairment: last 6 months.	At-work productivity; Absenteeism; Job control; Negative impact on worker identity (e.g., over-commitment); Beliefs about future work ability
Alipour (2013), Sweden [5]	Neck and/or back pain	Employees from Swedish workplaces (2 paper mills, 1 steelwork, 1 truck manufacturer).	1 stand-alone item.	Previous year	Absenteeism
Azevedo (2012), Portugal [7]	Chronic pain	Household survey of Portuguese adults.	Absenteeism: 1 stand-alone item.<br>General job impacts: 1 stand-alone item.	Absenteeism - Previous 6 months.<br>General job impacts due to pain- any time in the past.	Job adjustments; Change of job; Job loss; Absenteeism
Benyamina Douma (2017), Canada [10]	Low back pain	Police officers.	Impact on work (prevented from doing normal work): NMQ - 1 item.	Impact on work: Previous 12 months.<br>Job adjustment: Any time since onset of pain.	Job adjustments; Change of job; At-work productivity

			Job adjustment/Change of job or duties: NMQ - 1 item.		
Birke (2017), Denmark [11]	Chronic non-cancer pain	No information given.	Pain interference with work (Not specific so could be productivity decrease or sick leave & work encompassed work and work at home): SF-12 - 1 item.	Previous 4 weeks.	General impacts related to quantity and quality of work
Bosman (2019), Netherlands [13]	Low back pain	All employed and on certified sick leave	WAI	WAI: Various time periods depending on the item.	At-work productivity; Absenteeism; Beliefs about future work ability
Dawson (2011), Australia and New Zealand [18]	Low back pain	Employed nurses and midwives.	ERIQ - 6 items.	Current perceptions of over-commitment to work.	Negative psychosocial work impacts (e.g., work-related stress, job strain)
Dougados (2015), Europe, Asia and Latin America [21]	Pain related to nonradiographic axial spondyloarthritis (nr-axSpA)	Employed adults.	Presenteeism: WPAI:AS - 1 item.<br>Absenteeism: WPAI:AS - 2 items.<br>Overall work productivity loss (presenteeism plus absenteeism): WPAI:AS.<br>Work instability: RA-WIS.	WPAI: Past 7 days.<br>RA-WIS: Present time.	At-work productivity; Absenteeism; General impact on quantity and quality of work
Dutmer (2019), Netherlands [22]	Low back pain and/or leg pain	60% employed (including 32% who were on sick leave). 17% permanent work disability.	Work ability: WAS - 1 item.<br>Productivity: iPCQ - 5 items (3 - presenteeism + 2- absenteeism).	WAS: Present time (compared to lifetime best).<br>iPCQ (absenteeism and presenteeism): 4 weeks.	At-work productivity; Absenteeism
George (2010), USA [24]	Low back pain	56% employed, no other information (did not indicate if only those employed were asked the FABQ work questions).	FABQ-W – 7 items.	Current perception.	Work demands worsening pain; Fear avoidance/of movement
Glattacker (2018), Germany [25]	Low back pain	84.6% were employed at baseline. No other information.	Beliefs that work is responsible for pain - FABQ 1 item.	Current perception.	Work demands worsening pain; Fear avoidance/of movement

			Beliefs about return to work/work prognosis - FABQ - 1 item.		
Hara (2018), Norway [27]	MSK or other chronic pain	Sick-listed individuals on temporary work-disability benefits.	Beliefs about return to work/work prognosis: FABQ - 1 item.<br>Work self-efficacy: 1 stand-alone item.	FABQ: current.	Intentions about return to work; Self-efficacy
Heredia-Rizo (2019), Denmark [30]	Non-traumatic neck/ shoulder pain	Female computer users recruited through advertising at Aalborg University (minimum of 4 hours / day computer use).	DASH (work module) - 4 items.	Past week.	At-work productivity
Herman (2017), USA [31]	Low back pain	79% were employed.	Absenteeism: WPAI - 1 item.<br>Presenteeism: WPAI - 2 items.	WPAI: last 7 days.	At-work productivity; Absenteeism
Jay (2016), Denmark [35]	MSK pain in the neck, shoulders, upper and lower back, elbows and hands	Laboratory technicians	FABQ-W - 7 items.	Current perception.	Fear avoidance/of movement; Work demands worsening pain
Johansen (2013), Norway [36]	Neck pain	Outpatients with neck pain referred to an outpatient clinic. 60% on sick leave or disability-related pension; 75% worked in white-collar jobs, 25% in blue-collar jobs.	NDI (work section) - 1 item.	Present time	At-work productivity
Katajapuu (2019), Finland [39]	MSK pain	Setting: patients in an outpatient rehab clinic of a university hospital. No other work-related baseline information given.	WHODAS (work section) - 4 items.	Past 30 days	At-work productivity

Lacasse (2016), Canada [41]	Pain related to Fibromyalgia syndrome	Telephone interviews of patients. 45.6% had a paid job. Absenteeism measured in this subgroup.	1 stand-alone item.	Past 3 months	At-work productivity
Lipton (2020), Canada, the Czech Republic, Finland, Israel, Japan, Poland, Russia, Spain, and USA [42]	Chronic migraine	Only those who were employed completed the WPAI:GH.	Presenteeism: WPAI:GH - 1 item.<br>Absenteeism: WPAI:GH - 2 items.<br>Overall work productivity loss (absenteeism plus presenteeism) WPAI:GH - 3 items.	Previous 7 days.	At-work productivity; Absenteeism; General impact on quantity and quality of work
van Lunteren (2018), Netherlands, Norway and Italy [81]	Back pain	Setting: outpatient clinics. No baseline work/employment details given.	WPAI: GH - 3 items.	Previous 7 days.	General impact on quantity and quality of work
Mann (2016), USA [43]	HIV-related neuropathic pain	Setting: 12 community-based physician practices	WPAI: SHP customised for HIV-neuropathic pain - 3 items.	Previous 7 days.	At-work productivity
Mann (2013), USA [44]	Spinal cord injury-related neuropathic pain	Setting: 12 community-based physician practices. 13.6% employed for pay, 65% disabled (34.3% of these due to their neuropathic pain), 12.6% retired, 2.9% unemployed.	WPAI: SHP customised for spinal cord injury-related chronic pain - 3 items.	Previous 7 days.	At-work productivity
Mesas (2014), Spain [47]	Neck pain, low back pain, migraine and frequent headaches	All participants employed. Employment type: 26.8% executive, scientific and intellectual; 29.3% technical, office, and service professionals; 26% agricultural, operator and mechanical; 27.1% unskilled.	1 stand-alone item.	Previous 12 months.	Absenteeism

Mintken (2010), USA [48]	Shoulder pain	No baseline employment information was given except 11.3% of patients missed work as a result of shoulder pain	FABQ-W – 7 items.	Current perception.	Work demands worsening pain; Fear avoidance/of movement
Narayana (2015), USA [49]	Pain related to diagnoses of back pain, neuropathic pain, traumatic injury, osteoarthritis, fibromyalgia, cancer pain, or other chronic pain conditions	Employment: The two pain groups (with and without breakthrough pain) - overall 37.4% employed, 14.5% self-employed; 28.5% disabled	Presenteeism: SDS - 1 item; HPQ-SF - 1 item. Absenteeism: SDS - 1 item; HPQ-SF - 2 items.	Presenteeism: SDS: previous week; HPQ-SF: previous week and past 4 weeks/28 days. Absenteeism: SDS: Lost days previous week and previous year; HPQ-SF: Lost hours previous 4 weeks.	At-work productivity; Absenteeism
Osterhaus (2014), North America, Latin America and Europe [53]	Total and nocturnal spine pain in patients with axial spondyloarthritis	69.2% were employed and of those 33.5% had nonmanual jobs, 22.5% had mixed (manual and nonmanual) jobs, and 13.2% had manual jobs with no supervisory roles. 12.3% were unable to work due to arthritis. 5.5% were retired.	WPS - 1 item.	Previous month.	At-work productivity; Absenteeism
Perrot (2010), France and Germany [56]	Pain associated with fibromyalgia	35.5% employed FT or PT, 4.3% disabled and 26.1% retired	Interference with work: BPI - 1 item; FIQ - 1 item. Absenteeism/missed work (including housework): FIQ - 1 item.	BPI: Past 24 hours. FIQ: Past week.	At-work productivity; Absenteeism
Pokrzywinski (2019), Canada and USA [58]	Endometriosis-associated pain	Females employed FT or PT.	HRPQ - 2 items & HRPQ diary.	Past week.	Reduced working hours; At-work productivity; Absenteeism

Pokrzywinski[a] (2020), Canada and USA [59]	Endometriosis-associated pain	Females employed FT or PT in paid employment, and household work.	HRPQ - 2 items & HRPQ diary.	Past week.	Reduced working hours; At-work productivity; Absenteeism
Pokrzywinski[b] (2020), Canada and USA [60]	Endometriosis-associated pain	Females employed FT or PT in paid employment and household work.	HRPQ - 2 items & HRPQ diary.	Past week.	Reduced working hours; At-work productivity; Absenteeism
Rashid (2018), Sweden [61]	Neck / shoulder / back pain	Females (18 - 65 years of age); ≥ 50% sick leave of ≥1 month due to pain in the neck/shoulders and/or back for ≥3 months. Type of work: 70% blue-collar and 30% white-collar.	Work ability: WAI (all 7 items). Beliefs about return to work - 1 stand-alone item. Job strain: DCSQ - 10 items. Support at work**:** DCSQ - 6 items.	WAI: current. Beliefs about return to work: future 6 months. DCSQ: current.	At-work productivity; Intentions about return to work; (Low) Support from supervisors or employer; (Low) Support from colleagues; Negative psychosocial work impacts
Rostami (2014), Iran [63]	Low back pain	All participants employed; other employment information given	FABQ-W - 7 items.	Current.	Work demands worsening pain; Fear avoidance/of movement
Rothman (2013), Sweden [64]	Muscular pain (non-cancer)	21% unemployed, no other baseline employment baseline information.	SF-36 - 4 items.	SF-36: previous 4 weeks	At-work productivity
Roy (2013), USA [65]	Pain associated with work-related upper extremity injuries (shoulder/upper arm, forearm, elbow, wrist and hand pain)	All employed workers with 43% not working due to injuries/pain.	RA-WIS	Current.	At-work productivity
Rudwaleit (2011), Europe [66]	Nocturnal pain and total back pain associated with ankylosing spondylitis	WPAI-SHP questionnaire only completed by employed patients. No other baseline employment information given.	Presenteeism: WPAI:SHP (low back pain) - 1 item. Absenteeism: WPAI:SHP (low back pain) - 2 items. Overall work productivity loss (absenteeism plus	WPAI-SHP: past 7 days	At-work productivity; Absenteeism; General impact on quantity and quality of work

			presenteeism): WPAI:SHP (low back pain) - 3 items.		
Ruiz-Montero (2019), Spain, Belgium, Netherlands, and Sweden [68]	Pain associated with fibromyalgia	No baseline employment information. Females.	Pain interference with work: (Not specific so could be productivity decrease or sick leave & work encompassed work and housework): FIQ - 1 item.<br>Absenteeism/Missed work (including housework): FIQ – 1 item.	FIQ: Past week	Absenteeism; General impact on quantity and quality of work
Sadosky (2013), USA [70]	Low back pain	52% of patients were employed at least part time.	Presenteeism: WPAI: (specific for chronic low back pain) - 1 item.<br>Absenteeism): WPAI (specific for chronic low back pain) - 2 items.	WPAI: Previous 7 days	At-work productivity; Absenteeism
Saltychev (2018), Finland [71]	MSK pain	No baseline employment information.	Impact of pain on day-to-day work/school: WHODAS - 1 item	Past 30 days.	At-work productivity
Stockbridge (2015), USA [78]	Chronic pain-related interference	Participants were from a US population household survey.	SF-12 - 1 item.	Past 4 weeks.	General impact on quantity and quality of work
Suso-Ribera (2020), Spain [79]	A pain population attending a tertiary pain clinic	Approximately 24% of patients employed at the time of assessment. 50% of patients with chronic pain. stopped working as a result (early retirement, permanent disability or stopped working), 34.2% no change in employment status due to pain.	Change in occupational status – stand alone item.	Since pain onset.	Job adjustments; Change of job; Job loss
Trinderup (2018), Denmark [80]	Low back pain	Participants on sick leave (49.8%), or at risk of imminent sick leave.	FABQ-W - 7 items.	Current perception.	Work demands worsening pain; Fear avoidance/of movement

de Vries (2013), Netherlands [20]	Nonspecific MSK pain	Participants employed at least 20 h a week during the 12 months prior to the study with absence from work due to musculoskeletal pain not more than 5% of potential total working hours.	Work ability: WAI - 1 item.<br>Perceived future work ability: WAI - 1 item.<br>Work performance on days worked: HPQ - 1 item.<br>Sick leave: WAI - 1 item.	Work ability (WAI): current.<br>Perceived future work ability (WAI): 2 years.<br>Work performance (HPQ) - Previous 4 weeks.<br>Sick leave (WAI) - past 12 months.	At-work productivity; Absenteeism; Beliefs about future work ability
Young (2011), USA [83]	Low back pain	22.9% employed. 49.2% on disability or applying for disability.	Interference of pain on work - 1 stand-alone item.	Current.	At-work productivity

Abbreviations:
BPI: Brief Pain inventory
DASH: Disabilities of the arm, shoulder, and hand questionnaire
DCSQ: Demand Control Support Questionnaire
ERIQ: Effort-Reward Imbalance Questionnaire
FABQ-W: Fear Avoidance Beliefs Questionnaire (work)
FIQ: Fibromyalgia Impact Questionnaire
HPQ: WHO Health & Work Performance Questionnaire
HPQ-SF: WHO Health & Work Performance Questionnaire-short form
HRPQ: Health Related Productivity Questionnaire
iPCQ: Productivity Cost Questionnaire
MSK: musculoskeletal
NDI: Neck Disability Index
NMQ: Nordic Musculoskeletal Questionnaire – Low back section (extended version)
RA-WIS: Work instability scale (Rheumatoid Arthritis)
SDS: Sheehan Disability Scale
SF-12, 36: The Short-Form Health Survey
WAI: Work ability index
WALS: Work Activity Limitations Scale, WALS
WHODAS: WHO Disability Assessment Schedule
WPAI (AS; GH; OH; SHP): Work Productivity and Activity Impairment questionnaire (Axial Spondyloarthritis; General Health; Osteoarthritis of the Knee or Hip; Specific Health Problem)
WPS: Work Productivity Survey

Supplementary File Table 3. Themes identified within qualitative studies exploring the impact of chronic pain on engagement in employment

First author, publication year, country*	Chronic pain context	Employment context (at baseline if longitudinal)	Concepts related to work identified as important within the context of chronic pain	
			**Themes**	**Subtheme: codes**
Ashby (2010), Australia [6]	Low back pain	All unemployed > 8 months. Previously employed as store workers, builder, self-employed roof tiler, labourer, butcher, furniture removals.	**Changes at work and to working status**	**Changes in employment**: Job loss<br>**Return to work**: Facilitators; Barriers; Intentions about return to work
			**Factors and impacts outside the work environment**	**Impacts**: Personal economic consequences
			**Psychological factors**	**Psychological impacts**: Negative impact on worker identity (e.g., overcommitment)<br>**Barriers to engagement in paid work**: Fear avoidance/of movement
Bailly (2015), France [8]	Non-specific low back pain	64% employed, 20% on sick leave, 8% retired, 4% unemployed and 4% work disabled	**Changes at work and to working status**	**Impacts related to the quantity and quality of work**: At-work productivity (including presenteeism, work ability)
			**Aspects of the workplace and work relationships**	**Barriers to engagement in paid work**: Low support from supervisors or employer
			**Pain and other related symptoms at work**	**Symptoms and barriers to engagement in paid work**: Intermittent nature of pain
			**Psychological factors**	**Psychological impacts**: Negative psychosocial work impacts (e.g., work-related stress, job strain)<br>**Perceived benefits of work**: Valuing identity as a worker
Carroll (2013), Canada [15]	Chronic pain related to MSK work injuries	Not working at the time of the study – either receiving worker's compensation or disability benefits. Occupations at the time of injury include construction	**Aspects of the workplace and work relationships**	**Facilitators to engagement in paid work**: Support from colleagues

First author, publication year, country*	Chronic pain context	Employment context (at baseline if longitudinal)	Concepts related to work identified as important within the context of chronic pain	
			Themes	Subtheme: codes
		workers, heavy labourer, skilled labourer service sector, and transport worker.		
Coutu (2011), Canada [17]	Chronic pain related to MSK work injuries	Employed but on prolonged sick leave. Mix of white- and blue-collar workers.	**Changes at work and to working status**	**Return to work**: Facilitators
			**Aspects of the workplace and work relationships**	**Barriers:** Low support from supervisors or employer
			**Psychological factors**	**Psychological impacts**: (Expected) impact of being unemployed<br>**Perceived benefits of work**: Sense of purpose and value
Glavare (2012), Sweden [26]	MSK pain related to whiplash, fibromyalgia, nerve injury and arthrosis (osteoarthritis)	73% employed. Occupations (including before unemployment for those unemployed) included business owners (transport, shop and home help), shop assistants, car mechanic, cleaner, watchman, office clerk, and dental nurse.	**Changes at work and to working status**	**Return to work**: Barriers
			**Aspects of the workplace and work relationships**	**Facilitators to engagement in paid work**: Reducing physical work demands<br>**Barriers to engagement in paid work**: Issues with disclosure<br>**Perceived benefits of work**: Work providing structure and routine
			**Psychological factors**	**Psychological impacts**: (Expected) impact of being unemployed<br>**Facilitators to engagement in paid work**: Self-efficacy; Positive approach towards work<br>**Perceived benefits of work**: Sense of purpose and value
	Fibromyalgia	Employed females. Types of occupations: administration, caring	**Changes at work and to working status**	**Changes in employment**: Change of job

First author, publication year, country*	Chronic pain context	Employment context (at baseline if longitudinal)	Concepts related to work identified as important within the context of chronic pain	
			**Themes**	**Subtheme: codes**
Mannerkorpi (2012), Sweden [45]		professions, laboratory-based, industry-based, sales and gardening.		**Impacts related to the quantity and quality of work**: Reduced working hours; At-work productivity (including presenteeism, work ability); Absenteeism
			**Aspects of the workplace and work relationships**	**Facilitators to engagement in paid work**: support from supervisors or employer; support from colleagues; job control; Reducing physical work demands<br>**Barriers to engagement in paid work**: Low support from supervisors or employer; Low job control; Issues with disclosure
			**Pain and other related symptoms at work**	**Symptoms and barriers to engagement in paid work**: Work demands worsening pain; Fatigue
			**Psychological factors**	**Psychological impacts**:), Emotional exhaustion; Cognitive issues (e.g., concentration, memory), Negative impact on worker identity (e.g., over-commitment; Negative psychosocial work impacts (e.g., work-related stress)<br>**Facilitators to engagement in paid work**: Positive attitude towards work
			**Factors and impacts outside the work environment**	**Impacts**: Personal economic consequences
McCluskey (2014), UK [46]	Low back pain	Disability benefit claimants with GP-determined 'unfit for work'.	**Changes at work and to working status**	**Return to Work**: Barriers

First author, publication year, country*	Chronic pain context	Employment context (at baseline if longitudinal)	Concepts related to work identified as important within the context of chronic pain	
			**Themes**	**Subtheme: codes**
Nilsen (2014), Norway [51]	Non-malignant chronic pain	Not working due to pain, receiving full or partial sick leave or incapacity benefit. Wanting to RTW.	**Changes at work and to working status**	**Changes in employment**: Change of job<br>**Return to Work**: Facilitators
			**Aspects of the workplace and work relationships**	**Facilitators to engagement in paid work**: Reducing physical work demands
			**Pain and other related symptoms at work**	**Symptoms and barriers to engagement in paid work**: Work demands worsening pain (physical); Intermittent nature of pain
			**Psychological factors**	**Psychological impacts**: Cognitive issues (e.g., concentration, memory); Negative impact on worker identity (e.g., over-commitment); (Expected) impact of being unemployed<br>**Facilitators to engagement in paid work**: Positive approach towards work<br>**Perceived benefits of work**: Valuing identity as worker
O'Hagan (2013), Canada [52]	Persistent pain related to MSK work injuries	All employed but on sickness absence and receiving worker's compensation. Occupations include trade, labourer, public protection, machine operator, transportation, and service sector.	**Changes at work and to working status**	**Impacts related to the quantity and quality of work**: Reduced working hours<br>**Return to work**: Facilitators, Barriers
			**Aspects of the workplace and work relationships**	**Facilitators to engagement in paid work**: Reducing physical work demands
			**Psychological factors**	**Facilitators to engagement in paid work**: Self-efficacy<br>**Barriers to engagement in paid work**: Fear avoidance/of movement<br>**Perceived benefits of work**: Valuing identity as worker

First author, publication year, country*	Chronic pain context	Employment context (at baseline if longitudinal)	Concepts related to work identified as important within the context of chronic pain	
			**Themes**	**Subtheme: codes**
Palstam (2013), Sweden [54]	Fibromyalgia	Females employed full- or part-time.	**Changes at work and to working status**	**Changes in employment**: Job adjustments, Change of job<br>**Impacts related to the quantity and quality of work**: Reduced working hours
			**Aspects of the workplace and work relationships**	**Facilitators to engagement in paid work**: Support from supervisors or employer; Support from colleagues; Reducing physical work demands<br>**Barriers to engagement in paid work**: Low support from supervisors or employer<br>**Perceived benefits of work**: Work providing structure and routine
			**Pain and other related symptoms at work**	**Symptoms and barriers to engagement in paid work**: Fatigue<br>**Perceived benefits of work**: Work as a distraction from pain
			**Psychological factors**	**Psychological impacts**: Negative impact on worker identity (e.g., over-commitment); Negative psychosocial impacts (e.g., work-related stress, job strain)<br>**Facilitators to engagement in paid work**: Self-efficacy, Positive approach towards work<br>**Perceived benefits of work**: , Valuing identity as worker; Sense of purpose and value

First author, publication year, country*	Chronic pain context	Employment context (at baseline if longitudinal)	Concepts related to work identified as important within the context of chronic pain	
			**Themes**	**Subtheme: codes**
			**Factors and impacts outside the work environment**	**Impacts**: Personal economic consequences<br>**Facilitators to engagement in paid work**: Support from agencies/individuals outside the workplace
Persson (2011), Sweden [57]	Back, neck, hip, knee, shoulder, leg, fingers and wrist pain or chronic widespread pain	Past or present blue-or white-collar workers. Either working (33%), on sick leave (8%), receiving disability pension (33%), retired (17%) or unemployed (8%). Employment types included: local industry, trade, construction, healthcare, and agriculture.	**Changes at work and to working status**	**Changes in employment**: Job loss
			**Pain and other related symptoms at work**	**Perceived benefits of work**: Work as a distraction from pain
			**Psychological factors**	**Psychological impacts**: (Expected) impact of being unemployed
Rydstad (2010), Sweden [69]	Pain due to whiplash	Either unemployed or unable to work more than 50% of working hours due to pain. Professions include administration, sign language interpreter, laboratory assistant, locksmith, computer salesperson, accountant, teachers and network technician.	**Psychological factors**	**Psychological impacts**: (Expected) impact of being unemployed<br>**Facilitators to engagement in paid work**: Self-efficacy<br>**Perceived benefits of work**: Valuing identity as worker
			**Factors and impacts outside the work environment**	**Impacts**: Personal economic consequences
Silva (2011), Brazil [72]	Work-related MSK pain	On leave from work due to the condition or retired. Occupations included bank work, general services, welder, warehouse assistant, production assistant, office worker and a visual artist.	**Changes at work and to working status**	**Changes in employment**: Job loss<br>**Impacts related to the quantity and quality of work**: At-work productivity (including presenteeism, work ability); Absenteeism
			**Aspects of the workplace and work relationships**	**Facilitators to engagement in paid work**: Support from supervisors or employer; Job control

First author, publication year, country*	Chronic pain context	Employment context (at baseline if longitudinal)	Concepts related to work identified as important within the context of chronic pain	
			**Themes**	**Subtheme: codes**
			**Pain and other related symptoms at work**	**Perceived benefits of work**: Work as distraction from pain
			**Psychological factors**	**Psychological impacts**: (Expected) impact of being unemployed<br>**Facilitators to engagement in paid work**: Self-efficacy<br>**Perceived benefits of work**: Valuing identity as a worker; Sense of purpose and value
Soklaridis (2011), Canada [73]	Work-related MSK pain	Employed but not working and receiving benefits from the Workers Safety and Insurance Board.	**Psychological factors**	**Perceived benefits of work**: Valuing identity as a worker
Stinson (2013), Canada [77]	Chronic pain	No information.	**Changes at work and to working status**	**Changes in employment**: Job adjustments; Job loss<br>**Impacts related to the quantity and quality of work**: Absenteeism
Wainwright (2013), UK [82]	Chronic pain within the past year.	Employees, with the need of a sick- or fit-note in the previous 12 months or on current sick leave due to chronic pain. Type of occupations: teachers, academic, administrator, behaviour support assistant, contract manager, executive officer, Army major, manager, nurse, personal assistant, software developer/engineer.	**Changes at work and to working status**<br><br>**Aspects of the workplace and work relationships**	**Changes in employment**: Job adjustments<br>**Return to work**: Facilitators<br>**Facilitators to engagement in paid work**: Support form supervisors or employer; Support from colleagues; Reducing physical work demands

*Country relates to the country in which the participants resided

Supplementary File Table 4: Instruments and stand-alone items assessing the impact of chronic pain on work within each theme

**Instrument / Stand-alone Items** (relevant codes) (first author, publication year)	**Description of instrument / wording of stand-alone items**
**Changes at work and to working status**	
Changes in employment	
Single item assessing changes in occupational status* (*job adjustments, change of job, job loss*) (Suso-Ribera, 2020) [79]	Participants asked to choose one of the following options when asked about any changes to their job following pain onset: No change, early retirement, changed job, permanent disability, stopped working, increased working hours, reduced working hours, got fired, temporary sick leave, started working
Single item assessing changes in occupational status* (*job adjustments, change of job, job loss*) (Azevedo, 2012) [7]	Participants asked to choose one of the following options when asked about the consequences of pain on their job: *Job loss (fired/voluntary termination), change in job/professional responsibilities (e.g. time reduction, task changing), change of job or switching profession, early retirement, long term sick leave (≥ 3 months)*
Single item from the NMQ assessing changes in occupational status* (*job adjustments, change of job*) (Benyamina Douma, 2017) [10]	*Have you ever had to change jobs or duties (even temporarily) because of the trouble? No/Yes*
Ten items evaluating three categories of changes in employment* (*change of job*) (Agaliotis, 2017) [4]	Items include questions on: 1) occasional loss of work hours or work interruptions, 2) change in the type or nature of work, or 3) permanent changes of work hours or job
Impacts related to quantity and quality of work	
Single item assessing impacts relating to quantity of work* *(reduced working hours, absenteeism)*(Suso-Ribera, 2020) [79]	Participants asked to choose one of the following options when asked about any changes to their job following pain onset: No change, early retirement, changed job, permanent disability, stopped working, increased working hours, reduced working hours, got fired, temporary sick leave, started working
WALS (*at-work productivity*) (Agaliotis, 2017) [4]	Twelve items assessing workplace activity limitations due to chronic pain, physical problems, concentration, pace of work and working hours.
Single item assessing general at-work productivity loss (*at-work productivity*)(Azevedo, 2012) [7]	*Does your pain problem affect somehow your current job/occupation or the number of hours you work?* [*Yes,No*]
Single item from the HRPQ* (*reduced working hours*)(Pokrzywinski, 2019, 2020[a], 2020[b]) [58-60]	Participants asked to indicate which work impacts they had experienced due to illnesses (pain)/ treatments: forced to work part-time when they wanted to work full-time; kept them from having a job when they wanted to work full-time or part-time; or none of these options
Single item relating to interference of pain on work (*at-work productivity*) (Young, 2011) [83]	*How much does pain interfere with your normal work (including both housework and paid work)?* *[No effect/Small effect/Moderate Effect/Large Effect/Extreme Effect]*
Work section of the NDI assessing the ability to work (*at-work productivity*)(Johansen, 2013) [36]	Six questions about the amount of work that can be done, ranging from: I can do as much work as I want to / I can't do any work at all.
Single item from the BPI (*at-work productivity*) (Perrot, 2010) [56]	Participants asked to indicate how pain interfered with normal work (including both work and housework) in the past 24 hours [0-10 scale; does not interfere/completely interferes]

Single item in the daily 7-day diary (*at-work productivity*) (Agaliotis, 2013, 2017) [3,4]	*Your knee problems may affect your ability to work or perform daily activities. Please estimate your capacity for each day from 0% (unable to do usual work/activities) to 100% (fully functioning in usual role).*
Single item from the FIQ (*at-work productivity*)(Perrot, 2010; Ruiz-Montero, 2019) [56,68]	*When you worked, how much did pain or other symptoms of your fibromyalgia interfere with your ability to do your work, including housework? [1-10 scale; no problem with work-great difficulty with work]*
Two items to enable calculation of presenteeism lost hours (*at-work productivity*)(Herman, 2017) [31]	One item relating to proportion of time back pain affected productivity: participants asked to indicate how much low back pain affected productivity while working during the previous seven days [scale 0-10; problem had no effect on work/problem completely prevented me from working].<br>One item assessing hours worked: *During the past seven days, how many hours did you actually work?*<br>Presenteeism lost hours calculated by multiplying the proportion of time low back pain affected productivity by hours worked.
One item from the WPAI assessing % impairment while working (*at-work productivity*)(Lipton, 2020; Rudwaleit, 2011; Dougados, 2015; Sadosky, 2013; Herman, 2017) [21,31,42,66,70]	*Think about days you were limited in the amount or kind of work you could do, days you accomplished less than you would like, or days you could not do your work as carefully as usual. If health problems affected your work only a little, choose a low number. Choose a high number if health problems affected your work a great deal. Consider only how much health problems affected productivity while you were working. [scale 0-10; health problems had no effect on my work/health problems completely prevented me from working].* Calculation of percent impairment while working: (score/10) x 100
DASH work module (*at-work productivity*)(Heredia-Rizo, 2019) [30]	Four questions which ask about perceived difficulty in doing physical work-related tasks [1-5 scale; no difficulty-unable]
Three items from the HRPQ (*at-work productivity*) (Pokrzywinski, 2019, 2020[a], 2020[b]) [58-60]	Three measures of presenteeism. (1) Participants asked to indicate (a) number of hours of planned work per week (b) number of hours impacted by illness/treatments. Presenteeism as percentage of planned work lost per week calculated from (hours lost per week / hour planned per week) x 100.<br>(2) Participant asked to assess the percentage impact on work output in the previous week [scale of 0-100%; no impact – kept me from accomplishing anything]. (3) productive hours lost per year calculated from hours lost/week x 48 (USA)
Ten items evaluating three categories of changes in employment* (*at-work productivity, absenteeism*) (Agaliotis, 2017) [4]	Items include: 1) occasional loss of work hours or work interruptions, sick leave 2) change in the type or nature of work, or 3) permanent changes of work hours or job. [yes/no]
One item from the SF-12 (*at-work productivity*) (Stockbridge, 2015) [78]	*During the past 4 weeks, how much did pain interfere with your normal work (including both work outside the home and house work)? [Scale 1-5; extremely (1)- not at all (5)].*
Four items from SF-36 (*work ability*)(Rothman, 2013) [64]	Four items assessing work limitations due to physical health.
Single item from the WHODAS (*at-work productivity*) (Saltychev, 2018; Katajapuu, 2019) [39; 71]	*In the past 30 days, how much difficulty did you have in your day-to-day work? [1-5 scale; none-extreme or cannot do].*
Single item from the HPQ (*at-work productivity*) (Narayana, 2015; de Vries, 2013) [20,49]	*How would you rate your overall job performance on the days you worked during the last four weeks (28 days)? [0-10 scale; worst performance-top performance].*

Single item from the WPS assessing number of days with productivity < 50% (*at-work productivity*) (Osterhaus, 2014) [53]	*How many days in the last month was the patient's productivity at work reduced by half or more because of arthritis?*
Single item from the WPS assessing overall productivity loss (*at-work productivity*) (Osterhaus, 2014) [53]	*In the last month, how much has arthritis interfered with the patient's work productivity (work outside of home) [Scale 0-10; no interference (0) - complete interference (10)]*
iPCQ assessing productivity losses (*at-work productivity*)(Dutmer, 2019) [22]	Three items assessing number of working days affected by phsical or psychological problems when working and how much work could be done on those days [0-10 scale; not able to do anything-able to do as much as normal]
Single item from the SDS (*at-work productivity*) (Narayana, 2015) [49]	*The symptoms have disruptedyour work/school work [0-10 scale; not at all (0) – extremely (10)]*
WAI instrument assessing work ability (*at-work productivity*)(Bosman, 2019) [13]	The work ability index (WAI) was measured and summed to a workability score (range 7 to 49) according to WAI guidelines.
Single item from the WAI (*at-work productivity*) (Agaliotis, 2017; de Vries, 2013; Dutmer, 2019) [4,20,22]	Current work ability compared to highest work ability ever [0-10 scale; cannot work at all(0)- work ability at its best (10)].
Two items from the WAI assessing physical and metal demands of work (*at-work productivity*) (Agaliotis, 2017) [4]	Participants asked to rate current work ability with respect to physical and mental demands of work [1-5 scale; very poor (1)-very good (5)]
Single item from the NMQ (*at-work productivity*) (Benyamina Douma, 2017) [10]	*During the last 12 months have you been prevented from doing your normal work (at home or away from home) because of this trouble? [Yes/No].*
RA-WIS assessing work instability *(at-work productivity*)(Dougados, 2015; Roy, 2013) [21,65]	Set of 23 items which, taken together, assess the degree of 'mismatch' between an individual's functional ability at work and their work tasks [True/Not True]
Single item assessing changes in quantity of work* (*absenteeism*)(Azevedo, 2012) [7]	Participants asked to choose one of the following options when asked about the consequences of pain on their job: Job loss (fired/voluntary termination), change in job/professional responsibilities (e.g. time reduction, task changing), change of job or switching profession, early retirement, long term sick leave (≥ 3 months)
Single item assessing sick leave (*absenteeism*) (Alipour, 2013) [5]	*Have you been sick-listed due to back or neck pain during the past year? [yes, once; yes, several times; no]*
Single item assessing sickness absence (*absenteeism*) (Mesas, 2014) [47]	*In the last 12 months, were you absent from work due to a health problem? Consider all disease classes, health problems and/or injuries because of which you had to lose a day of work [No-Yes]*
Single item derived from the WPAI (*absenteeism*) (Agaliotis, 2013, 2017) [3,4]	*In the past 2 months, how many days off did you have due to your knee problems?*
Single item assessing work days lost (*absenteeism*) (Azevedo, 2012) [7]	*In the last six months, how many work days have you lost because of your pain problem? [No work days lost/1-5 lost work days /6-10 lost work days/11-15 lost work days/More than 15 lost work days]*
Single item from the FIQ (*absenteeism*) (Perrot, 2010; Ruiz-Montero, 2019) [56,68]	*How many days last week did you miss work, including housework, because of fibromyalgia? [0-7 scale]*

Single item from the WPAI (*absenteeism*) (Herman, 2017) [31]	*During the past seven days, how many hours did you miss from work because of low back pain? Include hours you missed on sick days, times you went in late, left early, etc., because of your low back pain.*
Two items from the WPAI assessing work time missed due to health (*absenteeism*) (Dougados, 2015; Lipton, 2020; Rudwaleit, 2011; Sadosky, 2013) [21,42,66,70]	(*1) During the past seven days, how many hours did you miss from work because of low back pain? Include hours you missed on sick days, times you went in late, left early, etc., because of your low back pain.*<br>*(2) During the past seven days, how many hours did you actually work?*<br>[Absenteeism expressed as percentage work time missed (Q1/(Q1+Q2)]
Single item from the SDS assessing days lost (*absenteeism*)(Narayana, 2015) [49]	*On how many days in the last week did your symptoms cause you to miss school or work or leave you unable to carry out your normal daily responsibilities?*
Two items from the HPQ-SF assessing hours lost (*absenteeism*)(Narayana, 2015) [49]	Participants asked to report how many hours their employers expected them to work in a typical week and the total hours worked in the previous four weeks. [Calculation of hours worked in the past 4 weeks.]
Single item from the WPS (*absenteeism*) (Osterhaus, 2014) [53]	*How many days in the last month did the patient miss work because of arthritis? [If none, please write 0]*
Two items from the HRPQ (*absenteeism*) (Pokrzywinski, 2019, 2020[a], 2020[b]) [58-60]	Participants asked to report how many work hours lost in the previous week due to illnesses/treatments and how many hours they were scheduled to work. [Absenteeism calculated as the percentage of planned work lost in previous week]
Single item from the WAI (*absenteeism*) (de Vries, 2013) [20]	*During the last 12 months how many whole days have you been off work because of illness? [1-5 scale; none/max 9 days/10-24 days/25-99 days/100-354 days]*
Single item assessing days lost due to pain (*absenteeism*) (Lacasse, 2016) [41]	*During the past 3 months, how many days have you been absent from work/have you had to cease your caregiving and household unpaid work because of your pain or pain-related medical visits?*
Single item from the iPCQ assessing costs of absenteeism (*absenteeism*) (Dutmer, 2019) [22]	Participants asked if they had been absent from work either in last 4 weeks due to illness [yes and number of days/no]. [Cost of absenteeism calculated for number of sick days extrapolated for 6 months and multiplied by the cost of production loss per hour for men or women]
Three items from the WPAI assessing both time missed form work due to health combined with the impact of health on work productivity (*general impact on quantity and quality of work*)(Dougados, 2015; Lipton, 2020; Mann, 2013, 2016; Rudwaleit, 2011; Sadosky, 2013; van Lunteren, 2018) [21, 42-44, 66,70,81]	*(1) During the past seven days, how many hours did you miss from work because of low back pain? Include hours you missed on sick days, times you went in late, left early, etc., because of your low back pain. Do not include time you missed to participate in this study.*<br>*(2) During the past seven days, how many hours did you actually work?*<br>*(3) During the past seven days, how much did your health problems affect your productivity while you were working? [0-10 scale; no effect – completely prevented work]*<br>*[Overall work productivity impairment calculated as Q1/(Q1+Q2)+[(1-(Q1/(Q1+Q2))x(Q3/10)]*100]*
Return to work	
Single item assessing beliefs about return to work (*Intentions about return to work*)(Rashid, 2018) [61]	*Do you believe you will return to the same work within 6 months?*<br>*[1-10 scale; 1 = highly unlikely (1)-highly likely (10)).*
Single item from the FABQ (*Intentions about return to work*)(Hara, 2018) [27]	*I do not think that I will be back in my ordinary work within three months. [0-6 scale; completely disagree-completely agree].*

Five items from the FABQ representing the work prognosis scale (*Intentions about return to work*) (Glattacker, 2018) [25]	Items include beliefs that the participant should not and cannot work with pain until it is treated, and beliefs of the likelihood of returning to work in the next 3 months, or never. [0-6 scale; completely disagree-completely agree]
**Aspects of the workplace and work relationships**	
Workplace, employer, and colleague facilitators	
Six items from the DCSQ assessing workplace support from supervisors and colleagues (*support from supervisors or employer; support from colleagues*) (Rashid, 2018) [61]	Items included items about getting on with colleagues and supervisors, colleagues being supportive and understanding if they had had a bad day, and if there was a pleasant atmosphere at work. [1-4 scale, strongly agree-strongly disagree]
Single item (*job control, also low job control*) (Agaliotis, 2017) [4]	*How much influence do you have over the pace of work, decision making with task, decision making within unit or policies?* [Scoring: 1= very little, 2= Little, 3=A moderate amount, 4=Much, 5=Very much. Binary scoring: 1-3 = low job control; 4,5 = high job control]
Workplace, employer, and colleague barriers	
Ten items evaluating three categories of changes in employment* (*low support from supervisors/employer*) (Agaliotis, 2017) [4]	Items include: 1) occasional loss of work hours or work interruptions, 2) change in the type or nature of work including difficulties with managers or co-workers, or 3) permanent changes to work hours or job [yes/no]
Single item (*job control, ALSO low control*) (Agaliotis, 2017) [4]	*How much influence do you have over the pace of work, decision making with task, decision making within unit or policies?* [Scoring: 1= very little, 2= Little, 3=A moderate amount, 4=Much, 5=Very much. Binary scoring: 1-3 = low job control; 4,5 = high job control]
**Pain and related symptoms at work**	
Symptoms and Barriers to engagement in paid work	
Four items of the FABQ (*work demands worsening pain*) (Mintken, 2010) [48]	Items include beliefs about work causing the pain and making the pain worse [0-6 scale; completely disagree-completely agree]
Seven items of the FABQ (*work demands worsening pain*) (George, 2010; Jay, 2016; Mintken, 2010; Rostami, 2014; Trinderup, 2018) *NB: verbatim wording of these items differ between references [24,35, 48,63,80]*	Items include physical work demands making pain worse, work being too heavy, would make pain worse or cause damage and the belief that the participant should not work [0-6 scale; completely disagree-completely agree]
**Psychological factors**	
Psychological impacts	
One item assessing reasons for working through sickness (*negative impact on worker identity incl. over-commitment*) (Agaliotis, 2017) [4]	*What is the main reason you would work through a sickness?*<br>*Pressure of work; unfair to colleagues; prior booking/commitment; no cover; work ethic; or would not work through a sickness*

Six items from the ERIQ (*negative psychosocial work impacts (e.g. work-related stress, job strain)* (Dawson, 2011) [18]	Items about different aspects of being fixated on work and work problems, even when not at work. [Strongly disagree/disagree/agree/strongly agree]
One item from the WAI assessing future work ability expectations (*beliefs about future work ability*) (de Vries, 2013) [20]	Item estimating belief of ability to do current job with present state of health in two years' time [Unlikely – Not certain – Relatively certain]
One item assessing future work ability expectations (*beliefs about future work ability*) (Agaliotis, 2017) [4]	*Do you anticipate leaving your job within the next two years as a result of your knee problems?* No-Yes/Unsure.
DCSQ – 10 items (*negative psychosocial work impacts (e.g. work-related stress, job strain*) (Rashid, 2018) [61]	Items include questions about psychological demands of the job (5 questions) and degree of decision latitude (5 questions). 'Job strain' was assessed as a composite measure by creating a ratio of psychological demands against the degree of decision latitude.
Psychological facilitators	
Single item assessing work self-efficacy (*self efficacy*) (Hara, 2018) [27]	*How strong is your belief that you will cope with functioning in the ordinary work force? [0-10 scale; higher scores indicating higher self-efficacy].*
Psychological barriers	
One item from the FABQ assessing the belief that work caused pain (*fear avoidance/of movement (incl. beliefs about work as cause of pain)*) (Glattacker, 2018)[25]	*My pain was caused by my work or by an accident at work [0-6 scale; completely disagree- completely agree]*
Seven items from the FABQ (*fear avoidance/of movement (incl. beliefs about work as cause of pain*) (George, 2010; Jay, 2016; Rostami, 2014; Trinderup, 2018) *NB: verbatim wording of these items differ between references* [24,35,63,80]	Items include physical work demands making pain worse, work being too heavy, would make pain worse or cause damage and the belief that the participant should not work [0-6 scale; completely disagree-completely agree]

Abbreviations

BPI: Brief Pain inventory
DASH: Disabilities of the arm, shoulder, and hand questionnaire
DCSQ: Demand Control Support Questionnaire
ERIQ: Effort-Reward Imbalance Questionnaire
FABQ-W: Fear Avoidance Beliefs Questionnaire (work)
FIQ: Fibromyalgia Impact Questionnaire
HPQ: WHO Health & Work Performance Questionnaire
HPQ-SF: WHO Health & Work Performance Questionnaire-short form
HRPQ: Health Related Productivity Questionnaire
iPCQ: Productivity Cost Questionnaire
NDI: Neck Disability Index
NMQ: Nordic Musculoskeletal Questionnaire – Low back section (extended version) Arthritis
SF-12: The Short-Form Health Survey 12
SF-36: The Short-Form Health Survey 36
SDS: Sheehan Disability Scale
WAI: Work ability index
WALS: Work Activity Limitations Scale, WALS
WHODAS: WHO Disability Assessment Schedule
RA-WIS: Work Instability Scale (Rheumatoid Arthritis)
WPAI: Work Productivity and Activity Impairment questionnaire (customised for various conditions)
WPS: Work Productivity Survey
